# Serum from Antiphospholipid Syndrome Patients Downregulates Endothelial lncRNAs HIF1A-AS1 and OIP5-AS1

**DOI:** 10.3390/ijms27104562

**Published:** 2026-05-19

**Authors:** Luis M. Amezcua-Guerra, María G. Soberanes-García, Laura Barragán-Huerta, Yaneli Juárez-Vicuña, Adriana Miguel-Álvarez, Paloma Rodríguez, Araceli Páez, Felipe Massó, Luis Chávez-Sánchez, Wendy G. Vázquez-González, Angélica Vargas-Guerrero, Luis H. Silveira, Betania Mazón-González, Malinalli Brianza-Padilla

**Affiliations:** 1 Department of Immunology, Instituto Nacional de Cardiología Ignacio Chávez, Mexico City 14080, Mexico; 2 Health Care Department, Universidad Autónoma Metropolitana, Xochimilco, Mexico City 04960, Mexico; 3 School of Medicine, Universidad La Salle, Mexico City 14000, Mexico; 4 Department of Rheumatology, Instituto Nacional de Cardiología Ignacio Chávez, Mexico City 14080, Mexico; 5 UNAM/INC Translational Research Unit, Instituto Nacional de Cardiología Ignacio Chávez, Mexico City 14080, Mexico; 6 Unidad de Investigación Médica en Enfermedades Metabólicas del Hospital de Cardiología, Centro Médico Nacional Siglo XXI, Instituto Mexicano del Seguro Social, Mexico City 06720, Mexico; 7 Unidad de Investigación Médica en Inmunología del Hospital de Pediatría, Centro Médico Nacional Siglo XXI, Instituto Mexicano del Seguro Social, Mexico City 06720, Mexico; 8 Laboratorio Central de Epidemiología, División de Laboratorios Especializados, Centro Médico Nacional La Raza, Instituto Mexicano del Seguro Social, Mexico City 02990, Mexico; 9 Hospital San Ángel Inn Universidad, Mexico City 03330, Mexico

**Keywords:** antiphospholipid syndrome, lncRNA, epigenetics, HIF1A-AS1, OIP5-AS1

## Abstract

This study aimed to evaluate whether serum from patients with primary antiphospholipid syndrome (APS) is associated with changes in the expression of long non-coding RNAs (lncRNAs) in endothelial cells. Human umbilical vein endothelial cells (HUVECs) were cultured with serum from 12 female patients with APS or 8 age-matched healthy female controls. The expression levels of HIF1A-AS1, OIP5-AS1, and GAS5 were quantified by RT-qPCR. Exposure of HUVECs to APS serum was associated with reduced expression of HIF1A-AS1 and OIP5-AS1 compared with cells stimulated with control serum. The median HIF1A-AS1 expression levels were 0.08 a.u. (interquartile range, 0.06–0.10) in APS-stimulated cells versus 0.14 a.u. (0.08–0.16) in controls (*p* = 0.044). Likewise, OIP5-AS1 levels were 0.09 a.u. (0.01–0.16) in APS-stimulated cells versus 2.24 a.u. (0.70–3.55) in controls (*p* = 0.018). In contrast, GAS5 expression did not differ significantly between groups (340 a.u. (310–3940 versus 358 a.u. (163–445); *p* = 0.290). In this proof-of-concept study, serum from APS patients was associated with selective downregulation of HIF1A-AS1 and OIP5-AS1 in endothelial cells. These findings support a potential link between circulating APS-related factors and endothelial lncRNA expression; however, no mechanistic or functional conclusions can be drawn.

## 1. Introduction

Antiphospholipid syndrome (APS) is a leading cause of acquired thrombophilia, accounting for approximately 10% of cases of stroke, myocardial infarction, and deep vein thrombosis [1]. Although the pathogenesis of APS remains incompletely understood, accumulating evidence indicates that antiphospholipid antibodies (aPLs) and other inflammatory mediators present in APS serum can chronically activate endothelial cells, thereby promoting a prothrombotic phenotype [2].

A central mechanism involves anti-β2-glycoprotein I (β2GPI) antibodies binding to β2GPI associated with phospholipids on the endothelial surface, thereby generating immunogenic neoepitopes. Oxidized β2GPI can further activate Toll-like receptor 4 (TLR4)-dependent pathways, triggering nuclear factor-kappa B (NF-κB) signaling and upregulating proinflammatory cytokines [3]. Such innate immune signaling may also influence epigenetic regulation, including the expression of long non-coding RNAs (lncRNAs), which are non-protein-coding transcripts longer than 200 nucleotides that regulate gene expression at the transcriptional, post-transcriptional, and chromatin levels [4].

Among these, the lncRNA Growth arrest-specific 5 (GAS5) is markedly downregulated in monocytes from patients with systemic lupus erythematosus (SLE), where it regulates inflammatory cytokine expression through the mitogen-activated protein kinase (MAPK) pathway and is suppressed by TLR4 stimulation with lipopolysaccharide (LPS) [5]. By analogy, components of APS serum that activate TLR4 may similarly modulate endothelial lncRNA transcription.

Other lncRNAs with vascular relevance include Hypoxia-inducible factor 1-alpha antisense RNA 1 (HIF1A-AS1), a regulator of endothelial survival. In human umbilical vein endothelial cells (HUVECs), silencing HIF1A-AS1 enhances proliferation, migration, and invasion while reducing apoptosis [6]. Likewise, Opa-interacting protein 5 antisense RNA 1 (OIP5-AS1) contributes to endothelial dysfunction and atherosclerosis. OIP5-AS1 is upregulated by oxidized low-density lipoprotein (oxLDL) in HUVECs, and its knockdown mitigates oxLDL-induced apoptosis and inflammation through the miR-98-5p/TLR4/NF-κB axis [7].

Given the proinflammatory and prothrombotic milieu characteristic of APS, circulating factors–including autoantibodies and inflammatory mediators–have been shown to influence endothelial cell activation and gene expression programs. Several signaling pathways, including those involving pattern recognition receptors and downstream transcriptional regulators, have been implicated in endothelial responses to inflammatory and thrombotic stimuli, although their specific contribution may vary depending on the experimental context.

Within this framework, we hypothesized that APS serum may modulate the expression of endothelial lncRNAs, including GAS5, HIF1A-AS1, and OIP5-AS1, with potential implications for endothelial activation and APS-associated vasculopathy. However, the present study was not designed to delineate the underlying signaling mechanisms. Rather, we conducted an in vitro proof-of-concept study to assess whether exposure to APS patient-derived serum is associated with changes in the expression of these lncRNAs in HUVECs.

## 2. Results

Twelve female patients with primary APS (median age, 33.5 years; IQR, 26.0–54.7), and eight age-matched healthy female controls (median age, 37 years; IQR, 30.0–39.7) were included. The clinical characteristics of the study population are summarized in Table 1.

Among APS patients, 66.6% had a history of obstetric morbidity, 50.0% had experienced arterial thrombosis, and 41.6% had a history of venous thromboembolism. Regarding their aPL profiles, 66.5% tested positive for anti-β2GPI IgG, 58.3% for lupus anticoagulant, and 35% for anticardiolipin IgG antibodies. Most patients (83.3%) were receiving oral anticoagulation, and 41.6% were on low-dose aspirin at the time of sample collection.

**Figure 1:**
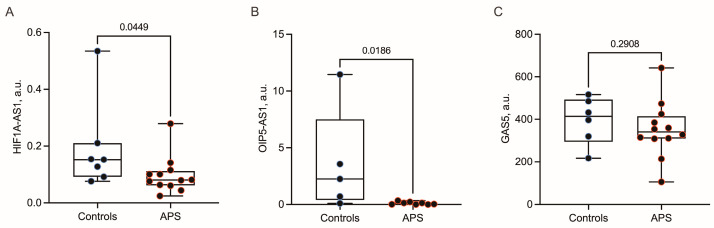
Effects of serum from patients with antiphospholipid syndrome (APS; red dots) and healthy controls (blue dots) on the expression of the long non-coding RNAs HIF1A-AS1 (**A**), OIP5-AS1 (**B**), and GAS5 (**C**) in human umbilical vein endothelial cells. Boxplots depict the median (horizontal line), interquartile range (box), and minimum and maximum values (whiskers). No data points were excluded as outliers.

Exposure of HUVECs to APS serum induced a marked downregulation of HIF1A-AS1 and OIP5-AS1 compared with cells stimulated with control serum (Figure 1A,B). Median HIF1A-AS1 expression levels were 0.08 a.u. (IQR, 0.06–0.10) in APS-stimulated cells versus 0.14 a.u. (IQR, 0.08–0.16) in controls (*p* = 0.044). Likewise, OIP5-AS1 levels were 0.09 a.u. (IQR, 0.01–0.16) in APS-stimulated cells versus 2.24 a.u. (IQR, 0.70–3.55) in controls (*p* = 0.018).

In contrast, GAS5 expression did not differ significantly between groups (Figure 1C). Median levels were 340 a.u. (IQR, 310–394) in APS-stimulated HUVECs versus 358 a.u. (IQR, 163–445) in control-stimulated cells (*p* = 0.290).

Additional analyses were conducted to explore potential differences in the expression levels of each lncRNA according to aPL positivity status, selected clinical manifestations (arterial thrombosis, venous thrombosis, and obstetric complications), and medication use, particularly heparins and antiplatelet agents. No statistically significant differences were observed in any of these comparisons.

## 3. Discussion

This study shows that exposure to serum from APS patients is associated with altered expression of selected lncRNAs in human endothelial cells, supporting a potential link between circulating APS-related factors and endothelial epigenetic regulation.

APS serum contains a complex mixture of autoantibodies, complement activation products, platelet-derived microparticles, and mediators of oxidative stress and hypoxia. These components may contribute to endothelial cell activation and gene expression through receptor-mediated mechanisms. Antiphospholipid antibodies, particularly anti-β2GPI, do not bind directly to membrane phospholipids but instead interact with β2GPI anchored to the endothelial surface through interactions with multiple receptors and co-receptors, including TLR2, TLR4, annexin A2, and members of the LDL receptor family such as LRP8 (ApoER2) and LRP6 [8]. These interactions occur within specialized membrane microdomains known as lipid rafts, which act as platforms for receptor clustering and signal transduction [9]. Although downstream pathways such as NF-κB activation and endothelial nitric oxide synthase inhibition have been described in this context [10], these mechanisms were not evaluated in the present study and therefore cannot be inferred from our findings.

HIF1A-AS1 has been described as a regulator of endothelial cell survival. In HUVECs, its silencing enhances proliferation, migration, and invasion while reducing apoptosis, whereas its overexpression induces cell-cycle arrest and apoptosis [6]. In addition, increased expression of HIF1A-AS1 has been reported under stress conditions such as hypoxia, where it has been linked to proapoptotic and proinflammatory responses [11]. In our study, exposure to APS serum was associated with reduced HIF1A-AS1 expression. While this observation is directionally consistent with previously reported functional effects of HIF1A-AS1, the present study did not assess endothelial cell phenotype. Therefore, no conclusions can be drawn regarding its impact on apoptosis, proliferation, or endothelial activation in this setting.

OIP5-AS1 is another stress-responsive lncRNA implicated in endothelial dysfunction. Prior studies have shown that OIP5-AS1 expression increases in endothelial cells exposed to oxLDL, promoting apoptosis and inflammation through mechanisms involving miR-98-5p and pathways linked to TLR4/NF-κB signaling [7,12]. In contrast, we observed a decrease in OIP5-AS1 expression following exposure to APS patient serum. This finding differs from reports in other experimental models [7,13,14]. The biological significance of this decrease cannot be established based on the current data, and no functional implications should be inferred.

Importantly, for both HIF1A-AS1 and OIP5-AS1, the present results are limited to expression changes and do not provide evidence of downstream functional effects or mechanistic pathways. Any interpretation regarding their role in endothelial activation, inflammation, or APS-associated vasculopathy should therefore be considered hypothesis-generating.

By contrast, GAS5 expression was not significantly altered by APS serum. GAS5 is a well-described regulator of apoptosis and inflammation [15], acting through both direct effects on immune and endothelial cells and indirect modulation of glucocorticoid receptor signaling [16]. In SLE patients, reduced GAS5 levels in monocytes correlate with increased IL-1β and IL-6 production via MAPK signaling [5]. Its stability in this study suggests that GAS5 may not be a primary target of APS-associated endothelial reprogramming, or that its regulation requires specific conditions, such as acute inflammatory flares or glucocorticoid exposure.

**Figure 2:**
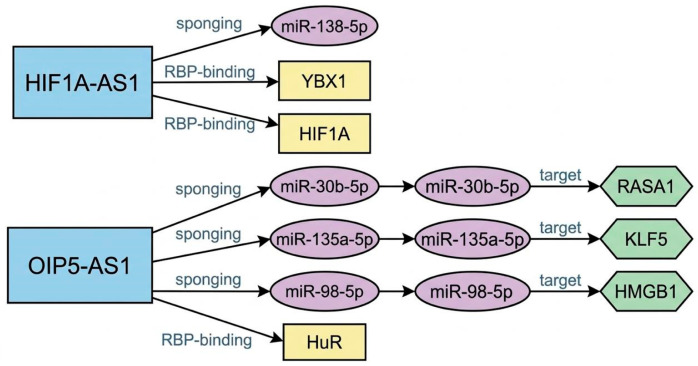
Conceptual and hypothetical interactome of the lncRNAs HIF1A-AS1 and OIP5-AS1 in endothelial cells, highlighting pathways potentially relevant to antiphospholipid syndrome. This schematic representation is based on previously reported interactions and is intended to provide a plausible biological framework; it is not derived from experimental validation in the present study. HIF1A-AS1 has been reported to interact with miR-138-5p via a sponging mechanism and to associate with the RNA-binding proteins YBX1 and HIF1A, suggesting a potential role in hypoxia-related regulatory processes. OIP5-AS1 has been described as a competing endogenous RNA capable of sponging miR-30b-5p, miR-135a-5p, and miR-98-5p, thereby modulating downstream targets such as RASA1, KLF5, and HMGB1, which have been implicated in inflammatory signaling pathways, including NF-κB activation. Additionally, OIP5-AS1 has been reported to interact with the RNA-binding protein HuR/ELAVL1, which stabilizes mRNAs encoding pro-inflammatory and pro-coagulant mediators such as tumor necrosis factor, interleukin-6, and tissue factor. Arrows denote reported or proposed regulatory interactions, including miRNA sponging and mRNA targeting.

To contextualize these findings, we constructed a simplified interactome integrating reported regulatory networks associated with HIF1A-AS1 and OIP5-AS1 (Figure 2). This model is intended to provide a conceptual overview based on prior literature and should not be interpreted as evidence of pathway activation in the present study.

This conceptual model is presented as a hypothesis-generating framework to contextualize the observed lncRNA expression changes and to identify candidate molecular pathways for future investigation.

Our study has several limitations that should be acknowledged. First, the relatively small sample size (*n* = 12 APS versus *n* = 8 controls) limits statistical power and may affect the robustness and generalizability of the findings. Although statistically significant differences were observed for selected lncRNAs, the magnitude of these differences and the associated p-values warrant cautious interpretation, particularly given the biological variability inherent to patient-derived serum. Second, the study design was cross-sectional and based on serum samples obtained during a quiescent phase of the disease. As such, the experimental conditions may not fully capture the dynamic inflammatory and prothrombotic milieu characteristic of active APS. Longitudinal studies and analyses under different clinical states would be necessary to better understand the temporal regulation of endothelial lncRNAs in this setting. Third, experiments were conducted using whole serum, which represents a complex and heterogeneous mixture of biological components, including autoantibodies, cytokines, complement factors, and microparticles. While this approach preserves physiological relevance, it precludes the identification of specific drivers of the observed effects and may contribute to inter-sample variability. In this context, it was not possible to isolate and evaluate the specific contribution of IgG fractions due to limited sample availability. Therefore, the observed changes in lncRNA expression likely reflect the combined influence of multiple circulating factors rather than a single defined component. Future studies using purified fractions and more controlled experimental systems will be required to dissect the relative contribution of individual mediators.

Importantly, this study was not designed to assess downstream functional effects or signaling mechanisms. Accordingly, the observed changes in HIF1A-AS1 and OIP5-AS1 expression cannot be linked to specific endothelial phenotypes, such as apoptosis, proliferation, or inflammatory activation. Any such interpretations should be considered hypothesis-generating and require direct experimental validation.

Despite these limitations, this study also has notable strengths. It evaluates the direct effect of patient-derived serum on human endothelial cells, providing a biologically relevant in vitro model that integrates the complexity of circulating APS-associated factors. In addition, the study focuses on lncRNAs with established vascular relevance, supporting a targeted and hypothesis-driven approach. The consistent directionality of the observed changes across independent lncRNAs suggests a non-random pattern of regulation in response to APS serum. Finally, our findings provide an initial framework for future investigation. While no mechanistic or functional conclusions can be drawn from the present data, the identification of differential lncRNA expression in response to APS serum supports further studies aimed at elucidating its role in endothelial biology. Given the emerging interest in RNA-based therapeutics, lncRNAs may represent potential targets [17]; however, their translational relevance in APS remains to be established through rigorous mechanistic and functional studies (Figure 2).

## 4. Materials and Methods

### 4.1. Participants

Twelve female patients with primary APS and eight age-matched healthy female controls were included in this study. Patients were recruited from the Rheumatology Clinic of the Instituto Nacional de Cardiología Ignacio Chávez (Mexico City, Mexico). Exclusion criteria comprised pregnancy, lactation, concomitant autoimmune disorders, malignancy, active infections, and end-stage organ failure. APS was diagnosed according to the revised 2006 Sidney criteria, and clinical classification was performed according to the 2023 ACR/EULAR criteria [18,19]. Fasting peripheral blood samples were obtained from all participants. Clinical and laboratory data were retrieved from medical records.

### 4.2. HUVEC Isolation and Culture

HUVECs were isolated from an umbilical cord segment obtained with written informed consent from a healthy 35-year-old woman undergoing elective cesarean delivery at Hospital San Ángel Inn Universidad (Mexico City, Mexico). The umbilical cord was rinsed to remove residual blood and transported in 30 mL of enriched DMEM medium (Sigma-Aldrich, St. Louis, MO, USA).

In the laboratory, the umbilical vein was flushed with sterile phosphate-buffered saline (PBS) to remove blood remnants. Endothelial cells were dissociated by perfusion with collagenase type II (1 mg/mL in PBS; Gibco, Grand Island, NY, USA), collected, washed, and cultured in M-199 medium supplemented with Earle’s salts, L-glutamine, 25 mM HEPES, 10% fetal bovine serum (Corning, New York, NY, USA), antibiotic-antimycotic solution (Gibco), and 1% endothelial cell growth supplement (Merck, Darmstadt, Germany). Cultures were maintained at 37 °C in a humidified incubator with 5% CO_2_. A culture medium-only control condition was included.

The culture medium was replaced daily to remove non-adherent cells. When cultures reached 80% confluence, cells were detached using trypsin-EDTA (Sigma-Aldrich) and resuspended for subsequent assays. HUVECs were used between passages 3 and 5. Endothelial cell identity was verified by flow cytometry using a PE-conjugated anti-human CD34 antibody (Clone 561; BioLegend, San Diego, CA, USA). For experimental procedures, 80,000 HUVECs were seeded per well in flat-bottomed 24-well plates (Costar, Corning, NY, USA) and cultured under the conditions described above. Cells were stimulated for 24 h with 10% serum derived from APS patients or healthy controls.

### 4.3. Quantitative RT-PCR for lncRNAs

Total RNA was extracted from cultured HUVECs, and complementary DNA (cDNA) was synthesized from 250 ng of RNA using the QuantiTect Reverse Transcription Kit (Qiagen, Hilden, Germany). Quantitative PCR was conducted on a CFX96 Real-Time PCR system (Bio-Rad Laboratories, Hercules, CA, USA) using the RT^2^ SYBR Green qPCR Master Mix (Qiagen).

Gene-specific primers were used to amplify the following lncRNAs: GAS5 (NR_002578.2), HIF1A-AS1 (ENST00000557544.0), and OIP5-AS1 (#LPH15948A-200_3316050). GAPDH (NM_002046.5) was used as the endogenous reference gene. Relative gene expression was calculated using the 2ΔΔCt method and reported as arbitrary units (a.u.).

### 4.4. Ethics Statement

The study protocol was approved by the Research Ethics Committee of the Instituto Nacional de Cardiología Ignacio Chávez (Approval No. 22-1304). Written informed consent was obtained from all participants. All procedures adhered to the principles of the Declaration of Helsinki and institutional guidelines.

### 4.5. Statistical Analysis

Data normality was assessed using the D’Agostino–Pearson omnibus test, which indicated a non-Gaussian distribution. Categorical variables were summarized as proportions and compared using Fisher’s exact test. Continuous variables were expressed as medians with interquartile ranges (IQR, 25th–75th percentiles) and analyzed with the Mann–Whitney U test.

All statistical tests were two-tailed, and significance was set at *p* < 0.05. Data analysis and graph generation were performed using GraphPad Prism version 10.3.1 (GraphPad Software, San Diego, CA, USA).

## 5. Conclusions

Serum from APS patients is associated with decreased expressions of HIF1A-AS1 and OIP5-AS1 in endothelial cells. These findings support a potential link between circulating APS-related factors and endothelial lncRNA regulation. However, given the descriptive nature of this study, no mechanistic or functional inferences can be drawn regarding their role in endothelial activation or thrombosis. Further studies are required to define the underlying mechanisms and to determine the biological and clinical relevance of these observations.

## Figures and Tables

**Table 1:** Clinical characteristics of study participants.

	APS (*n* = 12)	Controls (*n* = 8)	*p*-Value
Age, years	33.5 (26.0–54.7)	37 (30.0–39.7)	0.938
Body mass index, kg/m^2^	27.9 (24.3–29.5)	24.1 (22.9–25.7)	0.189
Comorbidities, *n* (%)			
• Hypertension	2 (16.6)	0	0.494
• Diabetes	2 (16.6)	0	0.494
• Dyslipidemia	1 (8.3)	0	>0.999
2023 ACR/EULAR clinical domains, *n* (%)			
• Macrovascular (venous thromboembolism)	5 (41.6)	-	
• Macrovascular (arterial thrombosis)	6 (50.0)	-	
• Microvascular	3 (25.0)	-	
• Obstetric	8 (66.6)	-	
• Cardiac valve	2 (16.6)	-	
• Hematologic	3 (25.0)	-	
2023 ACR/EULAR laboratory domains, *n* (%)			
• Lupus anticoagulant	7 (58.3)	-	
• IgG β2GPI	8 (66.6)	-	
• IgM β2GPI	1 (8.3)	-	
• IgA β2GPI	2 (16.6)	-	
• IgG aCL	7 (35.0)	-	
• IgM aCL	1 (8.3)	-	
• IgA aCL	0	-	
Pharmacological therapy, *n* (%)			
• Hydroxychloroquine	4 (33.3)	-	
• Rituximab	1 (8.3)	-	
• Oral anticoagulants	10 (83.3)	-	
• Aspirin	5 (41.6)	-	
• P2Y12 inhibitors	1 (8.3)	-	
• β-blockers	3 (25.0)	-	
• Calcium antagonists	1 (8.3)	-	
• ACEi/ARBs	4 (33.3)	-	
• Statins	3 (25.0)	-	
• Metformin	4 (33.3)	-	
• GLP-1 agonists	1 (8.3)	-	

Data are expressed as medians and interquartile ranges (25th–75th percentile), unless otherwise specified. Abbreviations: ACEi, angiotensin-converting enzyme inhibitors; aCL, anticardiolipin; ACR, American College of Rheumatology; APS, antiphospholipid syndrome; ARBs, angiotensin II receptor blockers; β2GPI, β2-glycoprotein I; EULAR, European Alliance of Associations for Rheumatology; GLP-1, glucagon-like peptide-1.

## Data Availability

The data presented in this study are available on reasonable request from the corresponding author. The data are not publicly available due to institutional and ethical restrictions related to patient confidentiality.
